# Surgical release of the chest wall skin and fascia for sclerodermatous graft versus host disease causing restrictive lung disease: A case report

**DOI:** 10.1016/j.ijscr.2024.109455

**Published:** 2024-03-07

**Authors:** Mitchell Nash, Kim Cartwright, Rebecca Nguyen, Peter Middleton, Peter Maitz

**Affiliations:** a Burns Unit, Concord Repatriation General Hospital, Concord, NSW 2137, Australia; b Department of Haematology, Wollongong Hospital, Wollongong, NSW 2500, Australia; c Department of Respiratory Medicine, Liverpool Hospital, Liverpool, NSW 2170, Australia; d South West Sydney Clinical School, Faculty of Medicine, University of New South Wales, Liverpool, NSW 2170, Australia; e Respiratory & Sleep Medicine, Westmead Clinical School, Westmead Hospital, Westmead, NSW 2145, Australia; f Sydney University, Camperdown, NSW 2006, Australia

**Keywords:** Case report, GVHD, Escharotomy, Restrictive lung disease

## Abstract

**Introduction:**

Graft versus host disease (GVHD) remains a significant source of morbidity and mortality in the setting of allogeneic stem cell transplantation. Skin involvement is reported to be as high as 70–95 % in this group with GVHD and the severity of the involvement varies widely. Surgical management of complications of severe cutaneous GVHD is uncommon and is rarely mentioned as a treatment option.

**Case presentation:**

We present a case of severe sclerodermatous skin changes restricting chest expansion and exercise tolerance to the point of limiting basic activities of daily life. A 54-year-old male presents with severe restrictive lung disease from sclerodermatous graft versus host disease (GVHD) after stem cell transplant for Chronic Myeloid Leukaemia (CML). He experienced limited symptomatic relief from maximal medical therapy and photochemotherapy, and subsequently underwent a skin release and split skin grafting of his chest and abdomen in an effort to improve exercise tolerance and quality of life.

**Clinical discussion:**

Despite an initial improvement in functioning, the patient's spirometry and lung function continued to decline with time, possibly suggesting that he did not gain a sustained benefit from surgical release of his cutaneous GVHD.

**Conclusion:**

While delineating between disease progression and surgical outcome is difficult in this case, the patient would argue that by delaying or reducing further decline in function, the surgical release procedures led to improved quality of life in subsequent years. However further research is required to establish a clear role for surgery in the treatment of refractory cutaneous GVHD.

## Highlights

• Surgical release with split skin grafting requires further evaluation to establish a clear role in the treatment of restrictive lung disease due to cutaneous GVHD
• Skin release (Chest Escharotomy Pattern) in restrictive lung disease caused by graft vs Host disease, objectively and subjectively improved the patient’s lung function.
• There were no adverse outcomes for the patient related to this surgical intervention, in fact the patient requested a second operative skin release and skin grafting.

## 1 Introduction

Graft versus host disease (GVHD) remains a significant source of morbidity and mortality in the setting of allogeneic stem cell transplantation [1]. Skin involvement is reported to be as high as 70–95 % in this group with GVHD and the severity of the involvement varies widely [2,3]. Severe skin involvement and the resultant scarring can lead to restrictive lung disease. Surgical management of complications of severe cutaneous GVHD is uncommon and is rarely mentioned as a treatment option [1,4,5]. Surgical management of cutaneous GVHD is controversial with conflicting evidence from a scarce number of Level IV studies [[6], [7], [8]]. This work has been reported in line with the scare criteria [9].

In De Novo Scleroderma, skin grafts from clinically normal skin to a sclerodermatous bed become sclerodermatous. This suggests that the skin changes are due to the underlying disrupted vascular bed with activated fibroblasts, otherwise a split skin graft taken from a normal donor site would heal as a normal skin graft, rather than take on the characteristics of sclerodermatous skin. In the reverse scenario, when sclerodermatous skin is grafted onto a normal bed, it remains sclerodermatous, suggesting that the skin changes are irreversible. Otherwise, improvement in skin quality would be expected of sclerodermatous skin grafted onto a normal bed. Sclerodermatous skin used as a split skin graft does take though and can allow closure of traumatic or chronic wounds as well as surgical wounds from skin release.

Escharotomies have long been a mainstay of treatment for circumferential deep burns which cause a torniquet effect and result in poor perfusion to the affected limb [10,11]. Chest escharotomies have been used to improve ventilation in patients with impaired chest expansion from full thickness chest burns [12].

## 2 Case presentation

A 54-year-old male presents with severe restrictive lung disease from sclerodermatous GVHD following matched sibling myeloablative allogenic stem cell transplant for Chronic Myeloid Leukaemia (CML) in 2001. This was conducted prior to subsidised tyrosine kinase inhibitors being available in Australia to patients with CML in chronic phase. He remained in remission following his allograft.

His other general medical history included obstructive sleep apnea on home CPAP, hypertension, gastroesophageal reflux, and osteoporosis. Pre-operative transthoracic echocardiogram was notable for mild-to-moderate impairment of left ventricular systolic function without significant valvular abnormalities. His medications at time of surgery are summarized in Table 1.

**Table 1:** Regular medications at time of surgery.

Tacrolimus 3 mg mane/2 mg nocte	Pregabalin 225 mg BD
Prednisone 20 mg daily	Pantoprazole 40 mg nocte
Trimethoprim/sulfamethoxazole 160/800 mg BD twice weekly	Citalopram 10 mg nocte
Azithromycin 250 mg BD	Folic acid 5 mg daily
Seretide (fluticasone/salmeterol) 250/25 μg 2 puffs BD	Magnesium aspartate 1500 mg BD
Felodipine 2.5 mg mane	Cholecalciferol 1000 units daily
Tramadol 50 mg TDS	Intravenous gammoglobulin 30 g monthly
Oxycontin 5 mg BD	Zoledronic acid 5 mg yearly

He developed grade 2–3 skin, gastrointestinal and hepatic acute GVHD which settled with high dose steroids and cyclosporin. Despite continued immunosuppression, he developed chronic extensive GVHD from approximately twelve months post-transplant, initially involving his liver, followed by inflammatory immune myositis, mono-neuritis multiplex, chronic inflammatory demyelinating polyneuropathy, with left phrenic nerve palsy and left hemidiaphragm paralysis. Sclerodermatous skin involvement was first noted three and a half years post-transplant.

Immunosuppressive therapies included steroids, cyclosporin, sirolimus, tacrolimus, rituximab, and romidepsin, with monthly intravenous gammaglobulin. He was managed with psoralen-ultraviolet A (PUVA) photochemotherapy, and due to ongoing deterioration, was subsequently commenced on extra-corporeal photopheresis, on twice monthly maintenance therapy prior to surgery.

Despite these treatments, he experienced symptomatic and clinical deterioration, with declining lung function tests, difficulty swallowing, weight loss and abdominal pain due to the constriction of his sclerodermatous GVHD. In the 18 months prior to surgery, he had 6 separate hospital admissions for acute-on-chronic respiratory failure in the setting of his interstitial pulmonary GVHD and CO_2_ retention; 3 of these admissions required high dependency unit (HDU) for non-invasive ventilation (NIV). Formal lung function tests performed 3.5 months pre-operatively were consistent with severe restrictive lung disease, with FEV1 0.86 L (23 % predicted), FVC 1.14 L (23 % predicted), and FEV1/FVC of 75 %.

He was referred to a Burns Unit, for consideration of skin and soft tissue release with split skin grafting, to possibly improve his restrictive lung disease. Although there was no clear precedent to this treatment, the patient was willing to undergo this surgery as his quality of his life had become severely limited by his disease, particularly in the preceding twelve months.

He underwent elective surgery on the 15th of October 2012 carried out by the senior author. He had an uncomplicated peri-operative stay, including a planned 24 h in intensive care post-operatively, intubated and ventilated. He was noted to a have a grade III larynx requiring two-operator intubation with video-laryngoscope support and a fibre-optic scope.

**Fig. 1:**
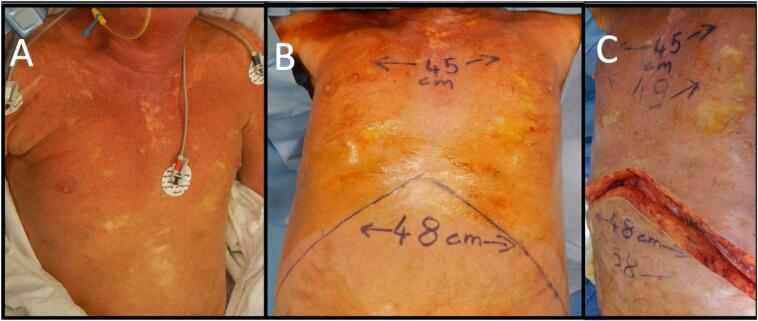
A: Skin appearance immediately pre-operatively. B: Chest and abdominal measurements prior to surgery. C: Measured increase on table post release and grafting.

**Fig. 2:**
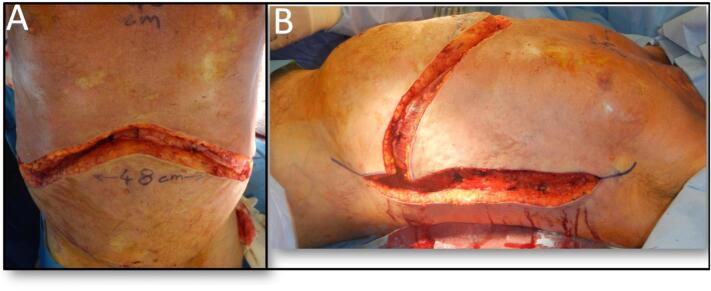
Depth of surgical release, through deep fascia exposing underlying musculature. A Subcostal. B Mid-axillary.

**Fig. 3:**
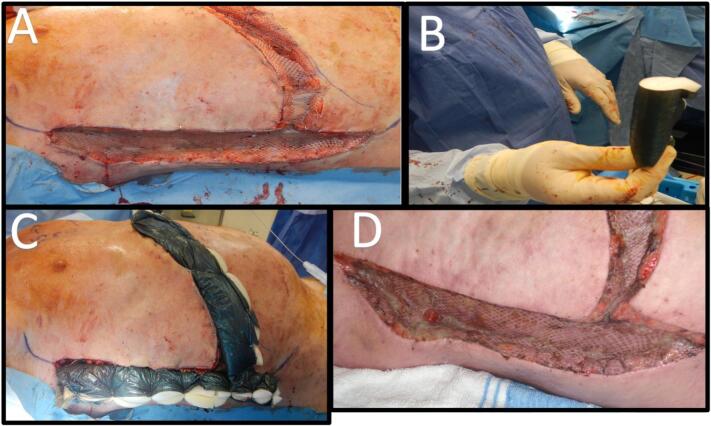
A: Intra-op post graft inset. B: Acticoat foam tube dressing C: Dressing in situ. D: Early (day 10) graft result.

At the time of surgery, he was noted to have widespread abnormal soft tissue (Fig. 1A) with extensive thick scarring (fascial-like bands) from the skin surface extending deep to his intercostal muscles. The pattern of escharotomy was anterior chest subcostal margin and mid-axillary (Fig. 2). The escharotomies were released from skin to deep facia, exposing serratus anterior, intercostals, external oblique and rectus muscles, using scalpel and diathermy. Foam rolled into a cylinder with Acticoat [Smith & Nephew] stapled on was used to deliver some compression on the vertical walls of the wound (Fig. 3B/C). Due to the location of the escharotomies, no postoperative splinting was feasible, and standard chest physiotherapy was performed. A sample of this tissue was sent for histopathology, reported as non-specific scar tissue and fibrosis. On table, he had a tidal volume increase of 100 mL, from 360 to 460 mL (PEEP 6cmH2O, Peak Airway pressure 21cmH2O). His chest transverse measurement (from midaxillary line) increased from 45 to 49 cm, and his abdominal girth increased from 48 to 58 cm (Fig. 1C).

**Fig. 4:**
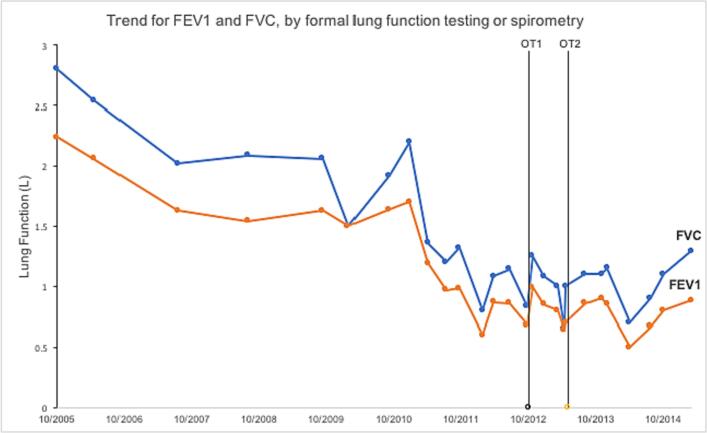
Trend for Forced Expiratory Volume-one second (FEV1) and Forced Vital Capacity (FVC), as measured by formal lung function testing or spirometry, over time, with dates marked for escharotomy and skin grafting of chest (OT1) and abdominal (OT2) wall.

Post-operatively, the patient described a subjective improvement in exercise tolerance and was able to return to enjoying lawn bowls. His informal spirometry improved significantly, from an FEV1 of 0.68 L, and FVC of 0.83 L pre-operatively, to 0.99 L and 1.25 L respectively post-operatively. Unfortunately, he did not undergo formal lung function testing immediately prior to surgery to allow for comparison. 9 months post-operatively, his lung function on spirometry had continued to regress, with an FEV1 of 0.7 L and FVC of 0.9 L, which was marginally better than his spirometry 4 months pre-operatively (Fig. 4).

**Fig. 5:**
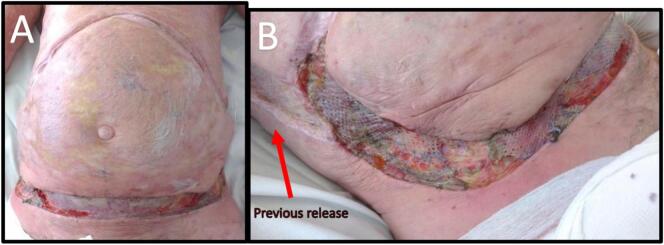
A: Further 10 cm transverse and 4 cm vertical skin release on table resulting in an increase of 50mls tidal volume. B: Note the maintained width of mature grafts from previous release.

The patient requested further skin release, and in May 2013 underwent an inferior transverse (supra-pubic) and longitudinal (meeting existing mid-axillary graft) abdominal skin release and split skin grafting (Fig. 5). His abdominal expansion increased 10 cm transversely and 4 cm longitudinally, with an immediate tidal volume increase of 50 mL. Informal spirometry showed a modest and transient improvement in FEV1 and FVC, from 0.64 L and 0.65 L pre-operatively, to 0.7 L and 1.0 L respectively post-operatively (Fig. 4). His spirometry results appeared to subsequently stabilise, although he was commenced on methotrexate in March 2014, which may potentially confound the benefits of his surgeries.

In support for the benefit surgical release, in his 9 admissions to hospital post-surgery, he did not require HDU for NIV, which is in contrast to the 18 months prior to his surgery where 3 out of 6 admissions required NIV. However, there was a clear downward trend of his lung diffusing capacity over time, reflecting the clear progression of his interstitial lung disease and the resulting decrease in function. The patient unfortunately passed away in December 2015, following a sudden cardiac arrest. An autopsy was not carried out and his death was presumed due to progression of his disease.

## 3 Discussion

Beredjiklian released joint contractures in the setting of cutaneous GVHD in 3 patients [8]. He found the released joints showed some initial improvement but relapsed to their pre-surgical range of motion. These releases involved a capsule release with or without muscle or tendon division/lengthening. No comment is made by the author specifying the incision or any skin lengthening procedures such as z plasties.

In contrast, a case report by Kim et al. released bilateral cubital fossa, axillae and shoulder contractures with multiple Y—V plasties. The underlying joints were normal and the improved range of motion was maintained at 9 months post-operatively [7].

Escharotomies of the chest are a standard management of full thickness burns restricting ventilation, as they may help relieve respiratory and hemodynamic dysfunctions [12]. Post-burn scar contractures which restrict movement are routinely released with split skin grafting with improved range of motion in combination with physiotherapy and splinting [12,13]. Thus, the premise that releasing thick chest wall scarring, with skin grafts should improve lung function and exercise tolerence in this case of cutaneous GVHD. However, despite initial improvement in hand-held spirometry results, formal lung function tests did not show any lasting objective improvement. A potentional confounder of the outcome of this surgery is ongoing progression of his interstitial pulmonary GVHD which had detoriated with time, in addition to the progression of his cutaneous GVHD.

The skin release objectively and subjectively improved the patient's function, given the objective improvement in lung function tests and the patient's request for further release surgery. This demonstrated that for this patient at least, they felt their quality of life was improved by the intervention and justified the hospital stay, pain and high-risk anaesthetic.

The authors conclude that there was no objective, sustained improvement in his lung function, although the decline in his lung function stabilised. Delineating between ongoing scarring and contraction of the split skin grafts (reducing their effect) versus ongoing disease progression is more difficult. Given the rarity of this presentation, we would suggest that the decision for surgery is best decided by a multi-disciplinary team approach in a case-by-case basis. Thus, surgical release and split skin grafting requires further evaluation to establish a clear role in the treatment of restrictive lung disease due to cutaneous GVHD. In this case escharotomy and skin grafting was a successful option to improve the quality of life of a patient and provide relief, at least temporarily, of the symptoms of restrictive lung disease. With appropriate informed consent, it is a potential treatment option for patients with this rare severe disease.

## Consent

Written informed consent was obtained from the patient for publication of this case report and accompanying images. A copy of the written consent is available for review by the Editor-in-Chief of this journal on request.

## Ethical approval

SYDNEY LOCAL HEALTH DISTRICT - CRGH.

HUMAN RESEARCH ETHICS COMMITTEE.

Approval No. CH 62/6/2015-215.

## Funding

No financial support or grants were received for this work.

## Author contribution

Prof Maitz – study concept and design, review of manuscript, Snr Author/Primary Surgeon.

Dr. Nash - Writing of manuscript (primary author)/data collection, assistant surgeon.

Prof Cartwright: editing and rewriting of manuscript, data analysis.

Dr. Nguyen: editing/review of manuscript, data collection, research.

Prof. Middleton: review of manuscript, data collection.

## Guarantor

Prof Maitz.

Dr. Kim Cartwright.

## Research registration number

N/A.

## Conflict of interest statement

Nil.
